# Preschool-level socio-economic deprivation in relation to emotional and behavioural problems among preschool children in Sweden

**DOI:** 10.1177/14034948231218040

**Published:** 2024-01-02

**Authors:** Natalie Durbeej, Richard Ssegonja, Raziye Salari, Anton Dahlberg, Helena Fabian, Anna Sarkadi

**Affiliations:** 1 Child Health and Parenting (CHAP), Department of Public Health and Caring Sciences, Uppsala University, Sweden; 2 Respiratory, Allergy and Sleep Research, Department of Medical Sciences, Uppsala University, Sweden

**Keywords:** Socio-economic deprivation, emotional and behavioural problems, preschool children, Strengths and Difficulties Questionnaire

## Abstract

**Aims:**

The aim of this study was to explore the association between preschool-level socio-economic deprivation and emotional and behavioural problems among preschool children in Sweden using a multilevel approach.

**Methods:**

In this cross-sectional study, we used data on 2267 children whose parents and preschool teachers had responded to items measuring individual-level socio-economic deprivation and the Strengths and Difficulties Questionnaire (SDQ) for assessment of emotional and behavioural problems. Further, the Socioeconomic Structure Compensation Index (SSCI), collected from Uppsala municipality, was used to assess preschool-level socio-economic deprivation. Unadjusted and adjusted multilevel logistic regression models were used to explore the relations between preschool-level socio-economic deprivation and emotional and behavioural problems.

**Results:**

In unadjusted models, children who attended preschools classified as highly deprived had elevated odds for emotional symptoms (odds ratio (OR) 1.71) as rated by parents. However, this association did not remain significant after adjusting for individual-level socio-economic deprivation factors. In both unadjusted and adjusted models, children who attended preschools classified as moderately deprived had elevated odds for peer-relationship problems as rated by parents (OR 1.63; adjusted OR 1.48). There were no significant associations between preschool deprivation and emotional and behavioural problems as rated by preschool teachers.

**Conclusions:**

**Swedish preschools may have a compensatory capacity in addressing children’s emotional and behavioural problems, whereas preschool-level deprivation remained significantly associated with peer-relationship problems after controlling for individual-level socio-economic deprivation factors. This implies that peer-relationship problems in deprived preschools need to be addressed in a broader community context.**

## Introduction

The preschool years play a critical role in life, as many skills and behaviours are formed during this period. Conditions that emerge during the preschool years not only affect the child directly but also affect future development and health [1]. Findings suggest that up to 30% of preschool children have mental-health problems [1,2]. Emotional and behavioural problems (e.g. sadness, anxiety, hypersensitivity, worry, hyperactivity and conduct problems) are the most common mental-health problems in preschool children and constitute a significant public-health concern [3]. These problems are likely to persist and be related to psychiatric disorders and increased treatment utilisation later in childhood [2,4]. The potential benefits of preventive interventions are significant, not only considering individual mental health but also as a future economic and social investment.

Due to the large proportion of time children spend at preschool and its widespread access to children at a critical stage of development, preschool has been highlighted as a key context for facilitating well-being and academic, social and emotional development, especially for socio-economically disadvantaged children [5]. Nonetheless, in Sweden, the quality of preschools has been found to be lower in disadvantaged socio-economic status (SES) areas – a circumstance that can affect future cognitive development and language skills negatively [5,6].

SES can be measured at various levels, for exam-ple individual, neighbourhood or school levels. Nei-ghbourhood- and school-level SES comprises measures at a group level, for example proportion of foreign-born children/inhabitants, living conditions, poverty rates, parental education levels and school grade average, whereas individual-level SES includes measures on an individual level such as parental education, occupation, income and marital status. Studies have found individual-level SES factors such as low parental education level, parental financial stress, immigrant status and parental unemployment to be associated with emotional and behavioural problems in preschool children and schoolchildren [7–10]. Additionally, in research on preschool children specifically, individual-level SES factors, including low income and low parental education level, have been related to increased treatment utilisation for mental-health problems later during childhood [11].

Studies have also found correlations between school-level SES and mental-health problems in young children. For instance, a Swedish study reported that a high proportion of foreign-born students and low school grade average were associated with externalising problems independently of individual-level SES variables among children in ninth grade [12]. Another study found that SES school deprivation measures, such as high proportions of children with one or both parents born abroad and low school grade average, were associated with increased suicide risk later in life. When controlling for individual variables, however, the risk was attenuated by approximately 50% [13].

In other recent research from the UK, a higher proportion of students eligible for free school meals, indicating school-level socio-economic deprivation, has been related to overall mental-health problems as well as externalising problems, independently of individual-level SES factors [14,15]. A scoping review concluded that school-level socio-economic deprivation exacerbated the negative effects of a low individual SES in relation to students’ mental-health problems but that favourable school characteristics, including good school climate and sport facilities, mitigated the negative impact of low individual SES on mental-health outcomes [16]. Overall, research on this topic highlights the importance of considering *both* individual- and school-level SES in relation to mental-health problems in young children.

Although socio-economic deprivation is associated with mental-health problems in childhood, it is not clear how measures of preschool-level socio-economic deprivation correlate with emotional and behavioural problems in preschool children. Addi-tionally, Swedish studies on the relationship between preschool children’s emotional and behavioural pro-blems and objectively collected socio-economic mea-sures are lacking.

### Aims

This study aimed to explore the association between preschool-level socio-economic deprivation and emotional and behavioural problems among preschool children in Sweden.

## Methods

### Study design

The current study utilised a cross-sectional study design with data from the Children and Parents in Focus project (the Focus study), a population-based study including children aged three to five years and for which data were collected in Uppsala, Sweden, between 2013 and 2017 [17]. In total, 7185 unique children from Uppsala municipality were included in the Focus study. The total participation rate was 48%. Given that the current study comprised variables on two levels (i.e. preschool-level (level 2) and individual level (level 1)), we utilised a multilevel approach to analyse the data (see below).

### Procedure

Parents of all children aged three to five years were recruited at participating child health centres (CHC; *n*=19) in connection with their child’s annual check-up visit. CHC nurses attached three sets of questionnaires to the invitation letter that was routinely sent home to each child before their annual visit. Parents were requested to fill in one questionnaire each and give the third questionnaire to the preschool to be filled in by the child’s teacher. Parents returned the completed questionnaires at their child’s CHC health check-up, and preschool teachers sent the questionnaire directly to the CHC in a prepaid envelope. Questionnaires included the Strengths and Diffi-culties Questionnaire (SDQ) for assessment of the child’s emotional and behavioural problems [18] and items on individual-level socio-economic deprivation. Additionally, the Socioeconomic Structure Co-mpensation Index (SSCI) from 2017, for assessment of preschool-level socio-economic deprivation, was collected from Uppsala municipality (see below) and merged with data from the Focus study.

### Participants

The participants of this study comprised 3714 unique children who were recruited during the Focus study in project years 2016–2017. Emotional and behavioural problems can be situational. Thus, a multi-informant approach has been recommended for assessing such problems in young children [18,19]. In this study, we used data from both parents and preschool teachers, providing more objective reports and reduced informant bias on the outcomes, as the teacher reports were also included. Only data from children with complete data on variables of interest from at least one parent *and* from the preschool teacher were included. If data were available from both parents, we randomly chose the data from one parent only.

Among the 3714 unique children, we excluded 266 children who attended a preschool with an undefined SSCI score (see below). In addition, 1120 children were excluded for whom no SDQ questionnaire from at least one parent and preschool teacher were available. Finally, another 61 children were excluded due to missing data on additional variables of interest (see below). Thus, 2267 unique children were included in the current study.

### Measures and variables

#### Preschool-level socio-economic deprivation (level 2)

The resource allocation to Uppsala municipality’s preschools is based on two factors: one general base amount that is a uniform compensation per child, and the Socioeconomic Structure Compensation (SSC), which is based on the socio-economic composition of children in each preschool [20]. The purpose of the SSC is to distribute economic resources based on children’s backgrounds and needs. By compensating for systematic socio-economic differences between preschools, the attending children get better prerequisites for attaining school goals later in life.

The SSC to preschools in Uppsala municipality is summarised yearly in the SSCI. This index is used to inform the municipality about the resource allocation to its preschools and is composed of statistics about parents’ socio-economic background on a group level, namely, immigration status, living conditions, economic support, highest level of education and the human development index. Based on the SSC, each preschool is assigned an individual score ranging from 32 to 400, with a mean set at 96.2 (*SD*=50.4). A higher score equals more preschool socio-economic deprivation and thus higher compensation. From Uppsala municipality, we collected the SSCI from 2017 to assess preschool-level socio-economic deprivation.

#### Emotional and behavioural problems (level 1)

Children’s emotional and behavioural problems were measured using the SDQ [18]. The SDQ is a 25-item questionnaire, available in both parent and teacher versions, covering the following subscales: emotional problems, conduct problems, peer-relationship problems, hyperactivity/inattention and prosocial behaviour. Each subscale score ranges from 0 to 10. Additionally, the four symptom subscales (all except prosocial behaviour) can be summed into total difficulties score ranging from 0 to 40. This composite score gives an overall indication of the child’s emotional and behavioural problems. Further, the SDQ total difficulties score has proven to be a good predictor for psychopathology [21]. Overall, the SDQ has demonstrated good construct validity, concurrent validity and internal consistency in previous research [19] and among preschool children within the Focus study [22,23].

#### Covariates (level 1)

We included the following variables measuring individual-level socio-economic de-privation in order to adjust for their potential confounding effect: parental country of birth (Sweden or other), parental marital status (married/cohabiting or single/living apart/other), level of parental education (not completed primary school/primary school, upper secondary school/training school or college/university for at least three years) and parental age when their first child was born (continuous variable). This decision was based on previous studies showing associations between emotional and behavioural problems and such factors in young children [10,24,25]. As our previous study on the Focus study data showed that fathers assessed emotional and behavioural problems to a slightly higher degree than mothers [23], we also adjusted the results for the responding parent’s sex (female or male).

### Statistical analyses

Means, standard deviations, frequencies and proportions were used for descriptive purposes, and chi-square and Fisher’s exact tests were used for comparisons between participant groups. The relations between preschool-level socio-economic deprivation and emotional and behavioural problems were investigated using unadjusted and adjusted multilevel logistic regression models, with parents and preschool teachers’ assessments analysed separately.

The main independent variable of the models was preschool-level socio-economic deprivation as measured through the SSCI and divided into three categories: low deprivation, set below the 75th percentile (SSCI scores 32–112); moderate deprivation, set at the 75th–89th percentile (SSCI scores 115–157); and high deprivation, set at the 90th percentile (SSCI scores ⩾159) of all preschools in Uppsala. These definitions were informed by previous research [26] and established in consultation with Uppsala municipality statistics department.

The outcomes were emotional and behavioural problems according to all the SDQ subscales and the total difficulties score, and were defined as scoring above or below the cut-off for the total score and each SDQ subscale, respectively. Sex- and age-specific Swedish cut-offs, based on data from the Focus study, were applied to establish whether the children had emotional and behavioural problems [27]. Only in cases when the main independent variable was significantly associated with the outcomes, as shown by the unadjusted models, was it tested further in adjusted models.

In the multilevel logistic regression models, we added covariates, as described above. Prior to running the models, data were checked for multicollinearity through variance inflation factor (VIF) values for all independent variables. VIF values ⩾10 indicate multicollinearity [28]. The VIF values for the independent variables in the study ranged from 1.01 to 1.10, indicating no multicollinearity. From the multilevel models, *p*-values, odds ratios (OR), 95% confidence intervals (CI) and Akaike Information Criterion values for model fit are reported. The analyses were conducted using IBM SPSS Statistics for Windows v26 (IBM Corp., Armonk, NY) and R v3.4.3 (The R Foundation for Statistical Computing, Vienna, Austria). *p*-Values < 0.05 were used to indicate statistical significance.

### Ethical considerations

Parents of all participating children gave their written informed consent on behalf of their children prior to inclusion in the study. The consent included authorisation for both parents and preschool teachers to provide information on the children. The study was approved by the Regional Ethical Review Board in Uppsala (document number 2012/437).

## Results

### Participant characteristics

The total sample (*N*=2267) was evenly distributed in terms of sex and age (Table I). Most parents were born in Sweden (83.0%), had a college/university degree (73.0%) and were married or cohabiting (94.0 %). Most children (*n*=1789) were attending preschools classified as low-deprivation preschools, whereas fewer were attending preschools classified as moderately deprived (*n*=366) or highly deprived (*n*=112). In total, the 2267 children in the study were attending 199 preschools located in Uppsala municipality, with a range of 1–46 children attending each preschool (not shown in Table I). Of 199 preschools in the study, 149 were classified as low-deprivation preschools, 34 as moderately deprived and 16 as highly deprived.

**Table I.** Background variables in the total sample, and participants attending preschools with low, moderate and high levels of socio-economic deprivation.

Variables	Total sample (*N*=2267)	Low deprivation (*N*=1789)	Moderate deprivation (*N*=366)	High deprivation (*N*=112)	*p* ^a^
	*n* (%)	*n* (%)	*n* (%)	*n* (%)	
Child age					n.s.
3 years	706 (31.1)	536 (30.0)	131 (35.8)	38 (34.8)	
4 years	802 (35.4)	642 (35.9)	122 (33.3)	39 (33.9)	
5 years	759 (33.5)	611 (34.2)	113 (30.9)	35 (31.3)	
Child sex					n.s.
Male	1149 (50.7)	897 (50.1)	189 (51.6)	63 (56.3)	
Female	1118 (49.3)	892 (49.9)	177 (48.4)	49 (43.8)	
Parental sex					n.s.
Female	1271 (56.1)	999 (55.8)	202 (55.2)	70 (62.5)	
Male	996 (43.9)	790 (44.2)	164 (44.8)	42 (37.5)	
Parental country of birth					***
Sweden	1882 (83.0)	1548 (86.5)	279 (76.2)	55 (49.1)	
Outside Sweden	385 (17.0)	241 (13.5)	87 (23.8)	57 (50.9)	
Parental age first child, *M* (*SD*, min–max)	30.8 (4.7, 15–56)	30.8 (4.6, 16–56)	30.8 (4.9, 15–49	29.8 (4.9, 18–44)	n.s.
Parental education level					**
Not completed compulsory school/compulsory school	39 (1.7)	23 (1.3)	11 (3.0)	5 (4.5)	
Gymnasium/training school	573 (25.3)	432 (24.1)	107 (29.2)	34 (30.4)	
College/university degree	1655 (73.0)	595 (74.6)	248 (67.8)	73 (65.2)	
Parental marital status					
Single/living apart/other^b^	137 (5.0)	80 (4.5)	45 (12.3)	12 (10.7)	***
Married/cohabiting	2130 (94.0)	1709 (95.5)	321 (87.7)	100 (89.3)	
Preschool index score, *M* (*SD*, min–max)	88.4 (35.6, 32–400)	74.2 (20.0, 32–112)	128.6 (11.2, 115–157)	184.3 (33.1, 161–400)	
Level of socio-economic deprivation					
Low deprivation (0-74^th^ percentile)	1789 (78.9)				
Moderate deprivation (75^th^-89^th^ percentile)	366 (16.1)				
High deprivation (⩾90^th^ percentile)	112 (4.9)				

a Chi-square/Fisher’s exact tests for comparisons between participants with low, moderate and high levels of socio-economic deprivation.

b For example, divorced or separated.

* *p*<0.05; **p<0.01; ***p<0.001.

High-deprivation preschools had larger proportions of children whose parents were born outside Sweden (50.9%) compared to preschools with low and moderate levels of socio-economic deprivation (13.5% and 23.8%, respectively; χ^2^ (2)=119.2, *p*<0.001; Table I). Further, preschools with high and moderate levels of socio-economic deprivation had larger proportions of children whose parents were single, living apart or having other marital status (10.7% and 12.3%, respectively) than low-deprivation preschools (4.5%; χ^2^ (2)=37.0, *p*<0.001). Additionally, preschools with high and moderate levels of socio-economic deprivation had smaller proportions of children whose parents had a college/university degree (65.2% and 67.8%, respectively) compared to low-deprivation preschools (74.6%; *p*=0.002).

Based on parents’ reports, preschools classified as having high and moderate levels of socio-economic deprivation had larger proportions of children with peer-relationship problems (25.0% and 24.3%, respectively) than preschools classified as low-deprivation preschools (16.5%; (χ^2^ (2)=16.0, *p*<0.001; Table II). Based on preschool teachers’ report, preschools classified as moderately deprived had a larger proportion of children with lack of prosocial behaviour (18.6%) than low- and high-deprivation preschools (13.6% and 9.0%, respectively; χ^2^ (2)=7.9, *p*=0.019).

**Table II.** Behavioural and emotional problems as measured by the Strengths and Difficulties Questionnaire (SDQ) in the total sample and participants attending preschools with low, moderate and high socio-economic deprivation.

Variables	Total sample (*N*=2267)		Low deprivation (*N*=1789)	Moderate deprivation (*N*=366)	High deprivation (*N*=112)	*p* ^a^
	*M* (*SD*)	*n* (%)	*n* (%)	*n* (%)	*n* (%)	
SDQ rated by parents						
Emotional symptoms	1.2 (1.3)	322 (14.2)	246 (13.8)	52 (14.2)	24 (21.4)	n.s.
Conduct problems	2.0 (1.7)	387 (17.1)	294 (16.4)	68 (18.6)	25 (22.3)	n.s
Hyperactivity/inattention	2.1 (2.0)	360 (15.9)	276 (15.4)	59 (16.1)	25 (22.3)	n.s.
Peer relationship problems	0.8 (1.3)	412 (18.2)	295 (16.5)	89 (24.3)	28 (25.0)	***
Lack of prosocial behaviour	8.3 (1.7)	306 (13.5)	233 (13.0)	57 (15.6)	16 (14.3)	n.s.
Total difficulties	6.1 (4.3)	246 (10.9)	185 (10.3)	44 (12.2)	17 (15.2)	n.s.
SDQ rated by preschool teachers						
Emotional symptoms	0.5 (1.0)	280 (12.4)	223 (12.5)	47 (12.8)	10 (8.9)	n.s.
Conduct problems	0.9 (1.5)	285 (12.6)	226 (12.6)	51 (13.9)	8 (7.1)	n.s.
Hyperactivity/Inattention	1.5 (2.1)	267 (11.8)	211 (11.8)	40 (10.9)	16 (14.3)	n.s.
Peer relationship problems	0.5 (1.1)	296 (13.1)	224 (12.5)	56 (15.3)	16 (14.3)	n.s.
Lack of prosocial behaviour	8.4 (2.1)	323 (14.2)	244 (13.6)	68 (18.6)	11 (9.8)	*
Total difficulties	3.4 (4.2)	213 (9.4)	162 (9.1)	42 (11.5)	9 (8.0)	n.s.

a Chi-square/Fisher’s exact tests for comparisons between participants with low, moderate and high levels of socio-economic deprivation.

* *p*<0.05; **p<0.01; ***p<0.001.

### Associations between preschool-level socio-economic deprivation and children’s emotional and behavioural problems

In the unadjusted models, children who attended preschools classified as highly deprived had elevated odds (OR 1.71) for emotional symptoms as rated by parents (Table III). Moreover, children who attended preschools classified as moderately (OR 1.63) and highly (OR 1.69) deprived had higher odds for peer-relationship problems as rated by parents compared to children attending preschools classified as having a low level of deprivation. In the adjusted models, the association between moderate preschool socio-economic deprivation and peer-relationship problems (OR 1.48) as rated by parents remained significant when controlling for covariates, but not the association between high socio-economic deprivation and peer-relationship problems or emotional symptoms. In terms of significant covariates, children whose parents had completed upper-secondary school or training school had elevated odds for emotional symptoms. Also, children who had parents born outside Sweden had elevated odds for both emotional symptoms and peer-relationship problems. There were no significant associations between preschool socio-economic deprivation and emotional and behavioural problems as rated by preschool teachers (Table IV).

**Table III.** Multilevel logistic regression analyses exploring the association between preschool-level socio-economic deprivation and child emotional and behavioural problems as rated by parents (*N*=2267).

Unadjusted model	Emotional symptoms	Conduct problems	Hyperactivity/inattention	Peer relationship problems	Lack of prosocial behavioir	Total difficulties
	OR (95% Cl)	OR (95% Cl)	OR (95% Cl)	OR (95% Cl)	OR (95% Cl)	OR (95% Cl)
Independent variables						
Intercept	0.16 (0.14–0.18)***	0.19 (0.17–0.22)***	0.18 (0.16–0.21)***	0.20 (0.17–0.22)***	0.14 (0.13–0.17)***	0.12 (0.10–0.13)***
Preschool socio-economic deprivation						
Low (ref.)						
Moderate	1.04 (0.75–1.43)	1.14 (0.83–1.58)	1.05 (0.77–1.44)	1.63 (1.24–2.13)***	1.23 (0.88–1.72)	1.18 (0.84–1.68)
High	1.71 (1.07–2.74)*	1.42 (0.85–2.36)	1.58 (0.98–2.54)	1.69 (1.08–2.64)*	1.12 (0.63–1.99)	1.55 (0.91–2.66)
AIC	1856.2	2075.1	1988.9	2141.9	1799.5	1562.0
Adjusted model	OR (95% Cl)	OR (95% Cl)	OR (95% Cl)	OR (95% Cl)	OR (95% Cl)	OR (95% Cl)
Independent variables						
Intercept	0.15 (0.06–0.36)***	–	–	0.10 (0.04–0.22)***	–	–
Preschool socio-economic deprivation						
Low (ref.)						
Moderate	0.95 (0.68–1.32)	–	–	1.48 (1.12–1.96)**	–	–
High	1.39 (0.85–2.28)	–	–	1.26 (0.79–2.10)	–	–
Parental age first child	1.00 (0.97–1.02)	–	–	1.02 (1.00–1.05)	–	–
Parental marital status						
Married/cohabiting (re.f)						
Single/living apart/other^a^	1.35 (0.86–2.13)	–	–	1.06 (0.68–1.65)	–	–
Parental education						
College/university (ref.)						
Upper secondary school/training school	1.36 (1.04–1.80)*	–	–	1.09 (0.84–1.40)	–	–
Not completed compulsory school/compulsory school	1.00 (0.41–2.46)	–	–	1.47 (0.71–3.01)	–	–
Parental country of birth						
Born in Sweden (ref.)						
Born outside Sweden	1.49 (1.10–2.01)**	–	–	2.18 (1.68–2.84)***	–	–
Parental sex						
Male (ref.)						
Female	1.10 (0.86–1.41)	–	–	0.84 (0.67–1.06)	–	–
AIC	1854.3	–	–	2111.7	–	–

Significant associations are marked in bold.

a For example, divorced or separated.

* *p*<0.05; **p<0.01; ***p<0.001.

OR: odds ratio; CI: confidence interval; AIC: Akaike Information Criterion.

**Table IV.** Multilevel logistic regression analyses exploring the association between preschool-level socio-economic deprivation and child emotional and behavioural problems as rated by preschool teachers (*N*=2267).

Unadjusted model	Emotional symptoms	Conduct problems	Hyperactivity/inattention	Peer relationship problems	Lack of prosocial behaviour^a^	Total difficulties
	OR (95% Cl)	OR (95% Cl)	OR (95% Cl)	OR (95% Cl)	OR (95% Cl)	OR (95% Cl)
Independent variables						
Intercept	0.13 (0.11–0.16)***	0.14 (0.12–0.17)***	0.12 (0.10–0.15)***	0.14 (0.11–0.16)***	**–**	0.10 (0.07–0.11)***
Preschool socio-economic deprivation						
Low (ref.)						
Moderate	1.07 (0.71–1.60)	1.12 (0.78–1.61)	0.93 (0.61–1.43)	1.30 (0.88–1.93)	**–**	1.34 (0.87–2.06)
High	0.71 (0.34–1.49)	0.53 (0.25–1.13)	1.34 (0.70-2.54)	1.23 (0.65–2.34)	**–**	0.90 (0.42–1.98)
AIC	1692.1	1716.5	1638.7	1751.5	**–**	1411.4

Significant associations are marked in bold.

a Due to large differences between comparison groups (high deprivation, *n*=11; moderate deprivation, *n*=68; and low deprivation, *n*=244), this outcome was excluded from the analysis to avoid biased results.

* *p*<0.05; **p<0.01; ***p<0.001.

OR: odds ratio; CI: confidence interval; AIC: Akaike Information Criterion.

## Discussion

### Associations between individual- versus neighbourhood-level socio-economic deprivation and children’s emotional and behavioural problems

According to the results from this study, children of parents with lower educational levels had increased risk for emotional symptoms, and children of parents born outside Sweden had an increased risk of both emotional symptoms and peer-relationship problems. Previous Swedish and international research, showing that low parental educational level and parental immigration status are associated with young children’s mental-health problems, supports these findings [7,25,29,30].

Further, moderate preschool-level socio-economic deprivation was associated with an increased risk for peer-relationship problems as rated by parents. These results remained significant when adjusting for covariates measuring individual-level socio-economic deprivation. Because most preschool-aged children in Sweden attend the preschool closest to their homes, especially in low SES areas where parental active choice of a preschool outside the local area is less common, the preschool SSCI can serve as a proxy for neighbourhood-level deprivation. With this consideration in mind, the observed association of preschool-level socio-economic deprivation and peer-relationship problems is in line with, for example, a Dutch study, showing that the effect of neighbourhood-level deprivation on preschool children’s behaviour problems, as rated by parents on the Child Behavior Checklist, remained significant after adjustment for individual level socio-economic deprivation [31]. Similar results were seen in a UK study, where neighbourhood-level deprivation was associated with peer problems at preschool age, as measured by the SDQ, but not with externalising and internalising problems, which were shown to be mediated by other factors [32]. Finally, a study in Glasgow showed differences in preschool teacher-rated child behaviour problems on the SDQ between neighbourhoods, even after adjusting for age, sex, area deprivation and year of school entry [33]. Thus, it would seem that neighbourhood as a factor affects child behaviour problems and specifically peer problems, even after controlling for individual-level socio-economic deprivation factors.

### Preschool as a compensatory arena

The importance of neighbourhood as a determinant of health is well known and has been a part of models attempting to understand health as a product of micro-, meso- and macrolevel factors [34]. In these determinants of health models, preschools are viewed as a crucial arena and part of the mesosystem. In 2021, roughly 513,000 children were enrolled in preschools in Sweden, which represents 86% of all the country’s one- to five-year-olds [35]. For three- to five-year-olds, the proportion was 95% enrolled, and the mean number of children per personnel was 5.1. Preschools are thus possibly the greatest compensatory agent in the welfare state, given the time children spend there and the internationally very advantageous child per personnel ratio, as well as a national early learning curriculum. The yearly cost of the preschool system is more than 80 billion SEK—in other words, a major investment for the welfare state.

In Sweden, preschools are explicitly tasked with compensatory action, meaning that they should compensate for individual disadvantage stemming from parental lack of resources and/or low SES [36]. More than half of municipalities in Sweden, including Uppsala municipality, use socio-economic weighting, such as the SSCI, for resource allocation to their preschools to compensate for the additional costs of providing more services to populations with less resources. Interestingly, as demonstrated by our results, there were no associations between preschool-level socio-economic deprivation and emotional and behavioural problems as rated by preschool teachers. These findings may have several explanations. For instance, emotional and behavioural problems can vary between the home and preschool contexts, causing differences between teachers’ and parents’ ratings [19]. We have also found in our previous studies that preschool teachers generally score lower on all SDQ subscales, providing less statistical variability in teacher scores [27]. Nevertheless, our results might also imply that preschools actually have a compensatory capacity in addressing children’s emotional and behavioural problems.

Children attending moderately deprived preschools had elevated risks for peer-relationship problems as rated by parents. This result suggests that the compensatory capacity on emotional and behavioural problems was not extended to peer problems, where an area effect remained significant. Whether this is due to local-area social cohesion, social capital, safety or other unobserved factors is not settled, but neighbourhood-level social deprivation certainly seems to create a need for compensatory interventions beyond the implications of individual disadvantage. For instance, mentoring programs along with skills and professional training for preschool teachers could be beneficial for dealing with diverse student populations and promoting children’s social skills [37,38]. In addition, interventions that address both the preschool and its local community context are most likely needed. A recent example from Norway demonstrated that addressing mental-health problems and disruptive behaviour in a lower-secondary school setting through also involving the local community in a dialogue and mutual learning impacted children’s contexts to reduce bullying, bad language and social exclusion, and improved cohesion and well-being at school while also impacting social inclusion in the local community [39]. Realising that welfare arenas do not act in isolation but in a local community context should impact the way the welfare state’s compensatory task is conceptualised. Rather than only focusing on attempting to compensate for individual disadvantage, creating inclusive communities around children and families should also be a target for compensatory action.

### Strengths and limitations

One limitation of this study refers to the cross-sectional study design. Thus, no causality can be established from the results. Further, the occurrence of individual-level SES factors in the sample differs from those of the Swedish general population. More specifically, the sample had higher proportions of parents with a high education level, with Swedish origin and who were cohabiting or married than the Swedish general population [40]. Also, most children attended preschools with a low level of socio-economic deprivation. More children from socio-economically deprived families and/or attending high-deprivation preschools might have given different results. Overall, generalisations of the results should be made with caution.

To the best of our knowledge, the SSCI has not been psychometrically evaluated. Thus, using this index to determine preschool-level socio-economic deprivation could be considered a limitation. In addition, the cut-off levels for defining low, moderate and high socio-economic deprivation could be considered arbitrary. Nonetheless, they were based on previous research on similar measures [26] and established in consultation with Uppsala municipality’s statistics department. This should be considered a strength. Furthermore, we did not have access to data on the amount each preschool received or how the amount was used. Thus, future research should explore such data in relation to emotional and behavioural problems in preschool children.

A strength is the use of the Swedish version of the SDQ for assessing emotional and behavioural problems, which previously has demonstrated good psychometric properties [19,27]. The main strength is the large population-based sample of preschool children rated by both preschool teachers and parents. Finally, given the lack of research that previously has explored the relation between preschool-level socio-economic deprivation and emotional and behavioural problems in preschool children in Sweden, our findings add knowledge on this topic.

## Conclusions

This study demonstrated no associations between preschool-level socio-economic deprivation and emotional and behavioural problems as rated by preschool teachers. Our findings imply that preschools might have a compensatory capacity in addressing children’s emotional and behavioural problems. On the contrary, we found associations between a moderate level of preschool socio-economic deprivation and peer relationship problems as rated by parents. This finding highlights the significance of neighbourhood-level social deprivation beyond the implications of indi-vidual disadvantage. Thus, preventive interventions, involving both the preschool as a compensatory arena and local communities, promoting peer relationship development and social inclusion are warranted.
